# Diverse definitions of the early course of schizophrenia—a targeted literature review

**DOI:** 10.1038/s41537-018-0063-7

**Published:** 2018-10-15

**Authors:** Richard Newton, Alice Rouleau, Anna-Greta Nylander, Jean-Yves Loze, Henrike K. Resemann, Sara Steeves, Benedicto Crespo-Facorro

**Affiliations:** 1 Austin Health, University of Melbourne, Melbourne, VIC Australia; 2 Lundbeck SAS, Paris, France; 3 H. Lundbeck A/S, Copenhagen, Denmark; 4 Otsuka Pharmaceutical Europe Ltd., Wexham, UK; 5 Costello Medical Consulting Ltd, Cambridge, UK; 6 Department of Medicine & Psychiatry, University Hospital Marqués de Valdecilla, IDIVAL, Centro de Investigación Biomédica en Red de Salud Mental (CIBERSAM), Santander, Spain; 7 Present Address: Peninsula Health, Frankston, VIC Australia

## Abstract

Schizophrenia is a debilitating psychiatric disorder and patients experience significant comorbidity, especially cognitive and psychosocial deficits, already at the onset of disease. Previous research suggests that treatment during the earlier stages of disease reduces disease burden, and that a longer time of untreated psychosis has a negative impact on treatment outcomes. A targeted literature review was conducted to gain insight into the definitions currently used to describe patients with a recent diagnosis of schizophrenia in the early course of disease (‘early’ schizophrenia). A total of 483 relevant English-language publications of clinical guidelines and studies were identified for inclusion after searches of MEDLINE, MEDLINE In-Process, relevant clinical trial databases and Google for records published between January 2005 and October 2015. The extracted data revealed a wide variety of terminology and definitions used to describe patients with ‘early’ or ‘recent-onset’ schizophrenia, with no apparent consensus. The most commonly used criteria to define patients with early schizophrenia included experience of their first episode of schizophrenia or disease duration of less than 1, 2 or 5 years. These varied definitions likely result in substantial disparities of patient populations between studies and variable population heterogeneity. Better agreement on the definition of early schizophrenia could aid interpretation and comparison of studies in this patient population and consensus on definitions should allow for better identification and management of schizophrenia patients in the early course of their disease.

## Introduction

Schizophrenia can be a debilitating psychiatric disorder, with the majority of patients experiencing significant comorbidity throughout the course of their illness. Studies have reported cognitive and psychosocial deficits already at the onset of disease,^1^ and a negative impact of longer time of untreated psychosis on treatment outcomes.^2,3^ Despite continued development of pharmacological, psychosocial and other treatment modalities over the last 5 decades, many patient outcomes often remain poorly managed, especially with regards to negative symptoms and cognitive impairment.^4^

Current evidence suggests that individuals with schizophrenia are not only at considerable psychosocial risks, but also at risk of biological harm that may be associated with relapse.^5^ Research has further proposed that treating patients during the earlier stages of schizophrenia reduces the likelihood or frequency of relapse, reduces disease burden and provides patients with more favourable outcomes, including in the long term.^3,6–8^

The lack of standardised definitions of early schizophrenia allows for considerable variation of patient groups between studies and limits analysis of comparative effectiveness of the interventions used. There is therefore an increasing interest for consensus regarding definitions of early stage disease. The aim of this targeted literature review was to map definitions currently used to describe patients with a recent diagnosis of schizophrenia who are early in the disease course (‘early course’ schizophrenia), as reported in clinical guidelines and as used in study inclusion criteria.

## Results

### Literature search and study characteristics

1845 articles from MEDLINE/MEDLINE in-process and 656 records from clinical trial registries were identified and reviewed for relevance. From these records, 466 primary studies used some form of early schizophrenia terminology. A total of 17 relevant treatment guidelines were identified and included in the review. Across all studies included in the review, a large proportion of studies were conducted in Europe (*n* = 177). Further, 66 studies from North America were identified, 19 studies from Australia and 204 studies from elsewhere in the world (Table 1). Most included studies were cross-sectional observational studies (*n* = 279), with further featured study designs including longitudinal observational studies (*n* = 92), randomised controlled trials (*n* = 50) and other interventional studies (*n* = 45).

**Table 1:** Summary of primary studies identified in the review

Region	Number of studies	Total number of participants	Publication years
North America	66	6722	2005–2015
Europe	177	37,180	2005–2015
Australia	19	1484	2005–2015
Other^a^	204	30,372	2005–2015

^a^ Other regions included Asia (*n* = 175), Middle East (*n* = 10), Central/South America (*n* = 8), Africa (*n* = 3) or encompassed multiple regions (*n* = 8)

### Terminology used in treatment guidelines

Most of the 17 identified treatment guidelines divided the patient population into those with a first episode of psychosis and those with recurrent episodes, and did not provide additional definitions of early schizophrenia.^9–25^ Three guidelines also included alternative terminology for patients with early schizophrenia as distinct from first-episode schizophrenia.^26–28^ In 2011, the American Psychiatric Association defined 'early course' as the period after recovery from a first episode of schizophrenia and extending up to the subsequent 5 years.^26^ Llorca et al. (2013) considered 'early course' to apply to patients who had been newly diagnosed with schizophrenia and who had had no previous antipsychotic treatment.^27^ The Schizophrenia Patient Outcomes Research Team (2010) used the term “recent onset schizophrenia”, but no associated definition was specified.^28^

### Definitions used in primary studies

**Fig. 1:**
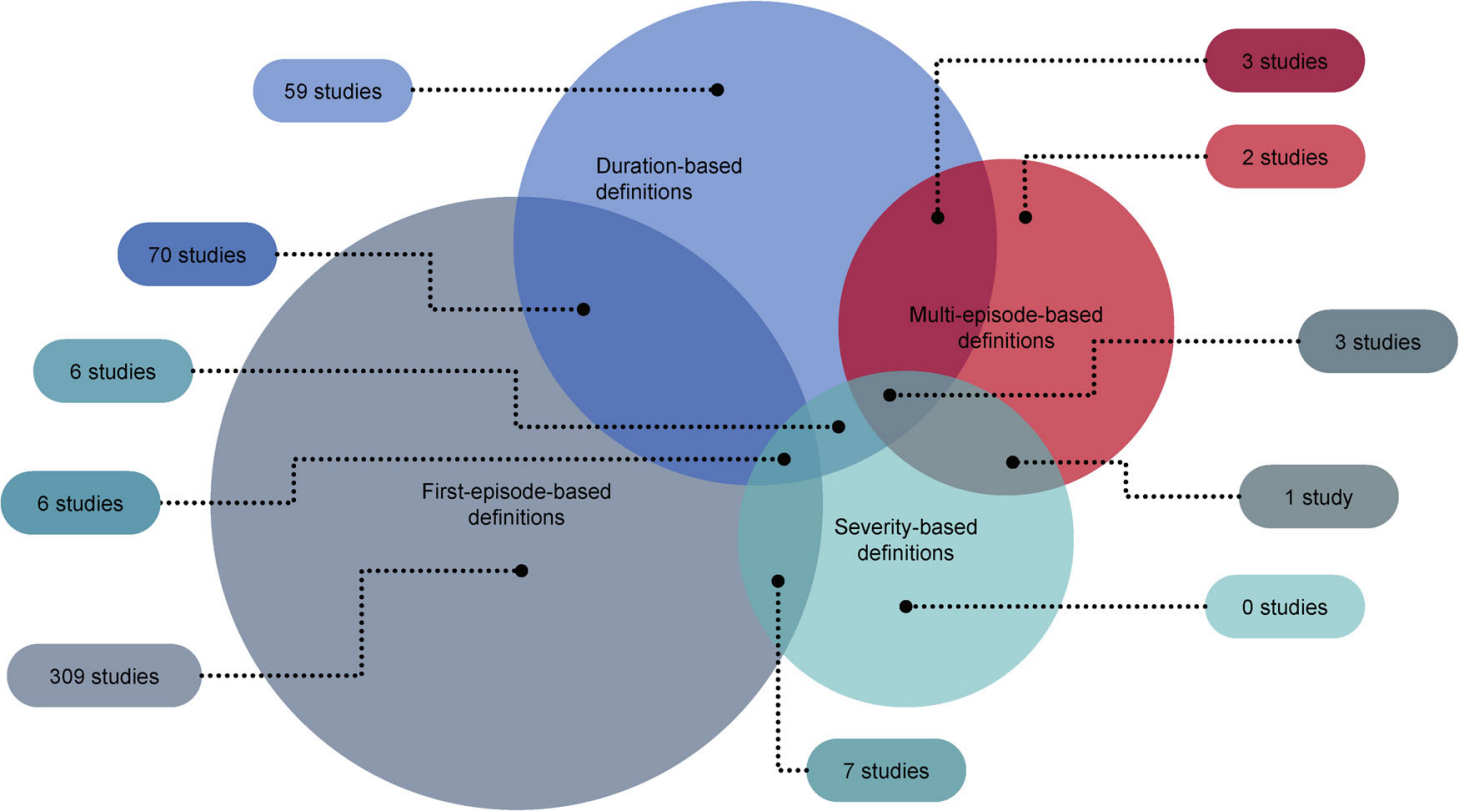
Summary of definitions used in primary studies (*N* = 466) identified in the literature review

Across the 466 primary studies using some form of early schizophrenia definition, three main definition types were identified (Fig. 1): episode-based definitions (*n* = 401), duration-based definitions (*n* = 147) and symptom severity-based definitions (*n* = 23). Episode-based definitions included those selecting patients on the basis of the number of psychotic episodes they have experienced. Duration-based definitions were definitions based on time since the start of illness, such as time since symptom onset, diagnosis or start of treatment. Symptom severity-based definitions included definitions based on current severity of symptoms and/or referring to the staging of schizophrenia as detailed in treatment guidelines.

**Fig. 2:**
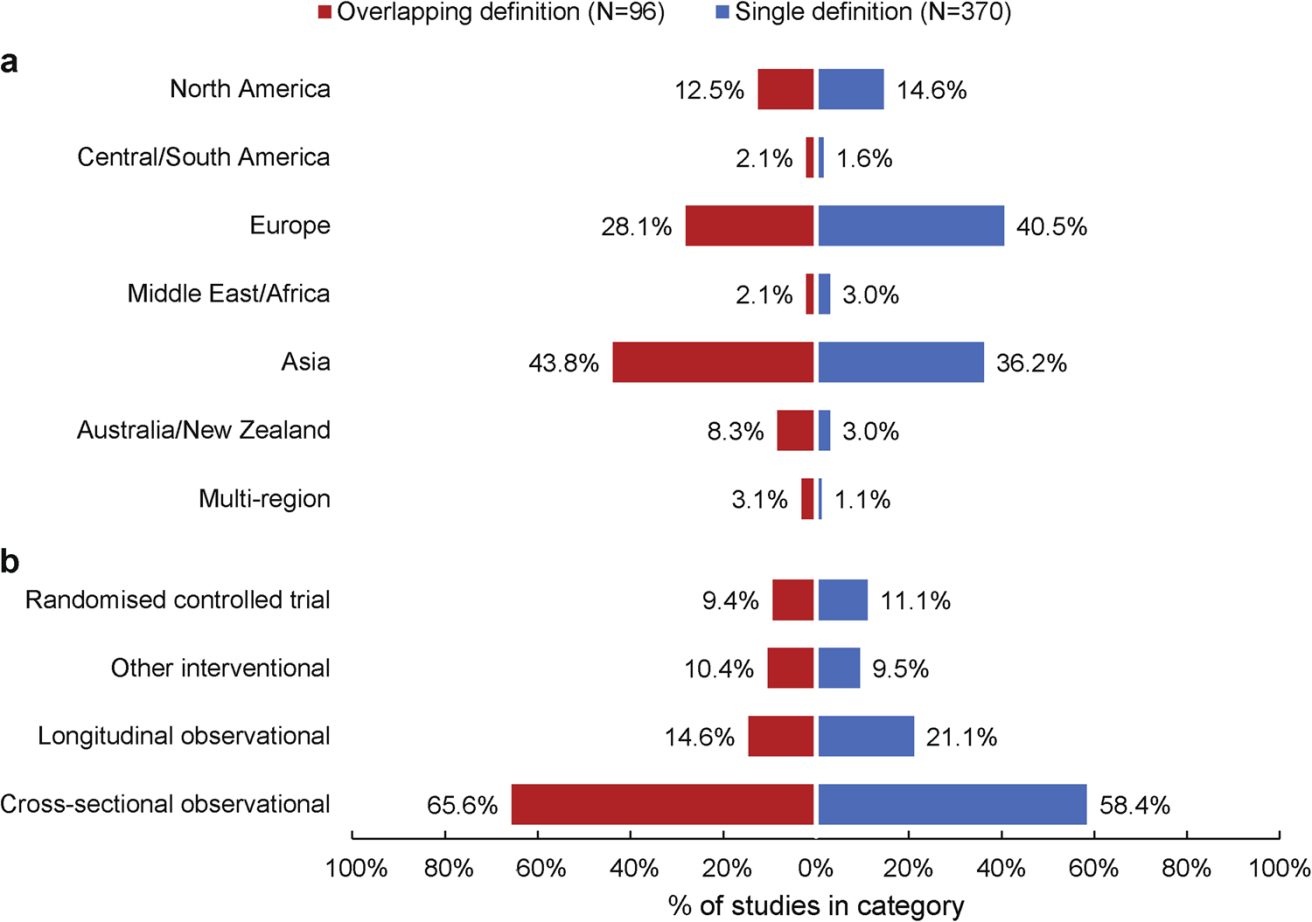
Proportion of studies (*N* = 466) using single vs overlapping early schizophrenia definitions; stratified by **a** study location and **b** design

There were 370 (79%) studies which used a single definition type, while the remaining 96 (21%) studies used overlapping definitions (e.g. first-episode and a duration-based definition). Stratifying these groups by study location revealed broadly similar patterns, with slightly higher proportions of European studies using single rather than overlapping definition types (Fig. 2). No substantial differences between the proportion of single and overlapping definitions were seen when stratifying by study design (Fig. 2).

#### Episode-based definitions

Of the 401 studies using episode-based definitions, 98% (*n* = 392) used ‘first-episode’ to define the enroled schizophrenia patient population. Nine studies did include multi-episode patients within a definition of early schizophrenia, but definitions were variable. Of these studies, three defined early schizophrenia in patients who had experienced <3 episodes.^29–31^ A further three studies selected early schizophrenia in patients who had experienced ≥1 episode with duration of illness ≤5 years,^32–34^ while an additional three studies defined early schizophrenia in patients who had experienced ≥2 episodes of relapse, again with an upper disease duration limit of 5 years,^35–37^ one of which required patients to have been hospitalised for those relapses.^37^

#### Duration-based definitions

Across primary studies using duration-based definitions to select patients, nearly half did not specify the disease onset definition used (71/147; 48%).^32,36,38–106^ The remaining 52% (*n* = 76) of studies used a variety of definitions for disease onset, including five studies that used multiple onset definitions: 29 studies referred to time since symptom onset;^107–135^ 14 studies referred to time since first episode or acute phase;^33,34,136–147^ 17 studies referred to time since first presentation to mental health services, hospitalisation or admission;^138,148–163^ 17 studies referred to time since schizophrenia diagnosis;^37,108,137,164–177^ and 5 studies referred to time since treatment had first been initiated.^35,108,109,149,178^

**Fig. 3:**
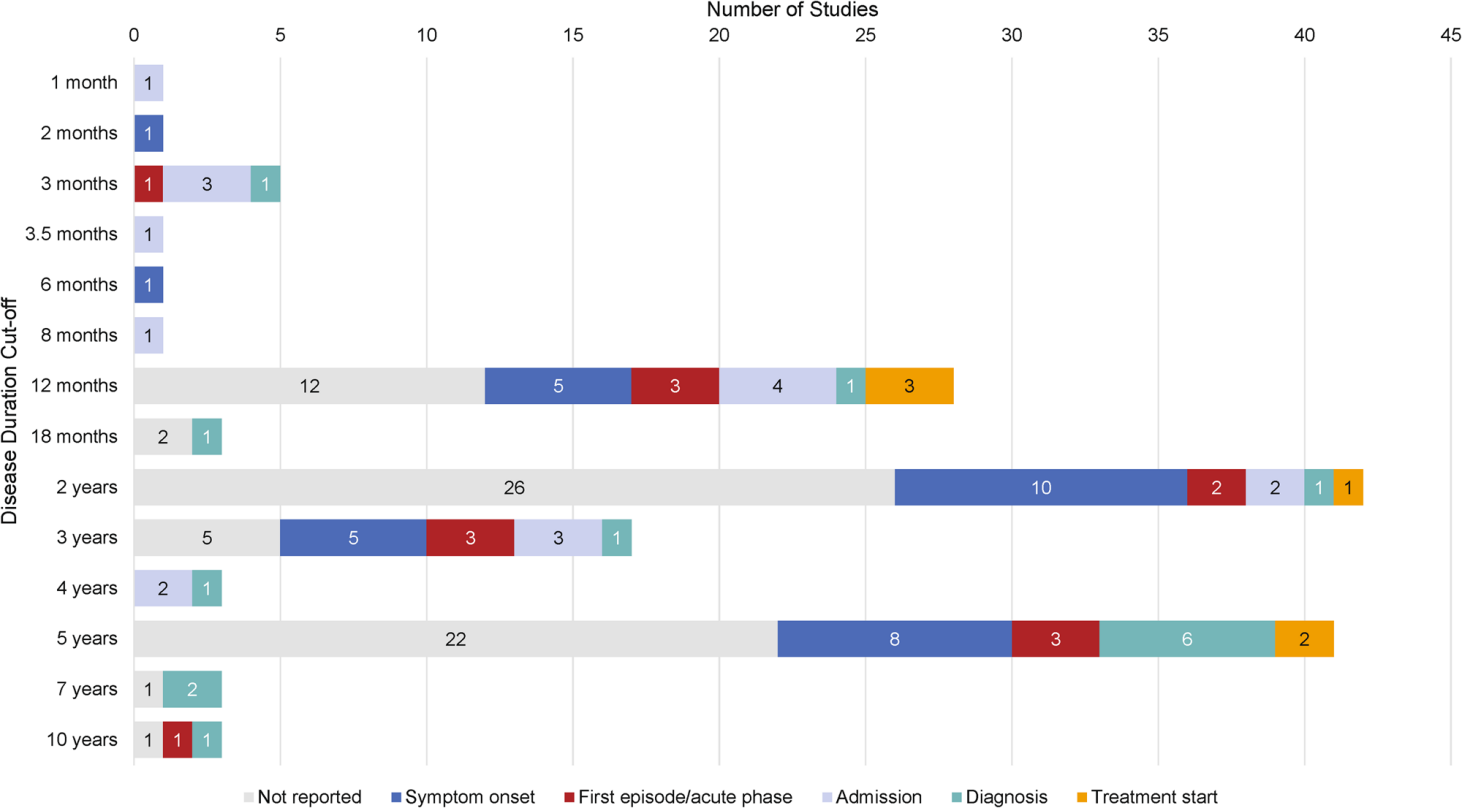
Distribution of cut-offs used with duration-based criteria, stratified by disease onset definition (*n*). Footnote: studies with multiple definitions were repeated under each category (*n* = 5)

In total, 142 of these studies selected patients with disease duration of less than a particular period of time to define early schizophrenia.^32–36,38–58,60–75,77–139,141–175,177,178^ Durations ranged from <1 month to <10 years, while 1, 2 or 5 years were the most common duration cut-offs used with duration-based criteria (Fig. 3). 91% (129/142) of studies did not provide a rationale for the duration cut-offs used. Of the remaining 9% (13/142) that did specify a rationale for their duration-based definitions, six established their definitions on prior experience and evidence,^34,90,93,150,152,164^ while seven studies provided study-specific rationale (Table 2).^32,42,45,52,63,154,169^

**Table 2:** Rationale provided in primary studies for the selection of patients with disease duration under a particular threshold

Rationale based on prior experience and evidence	Rationale linked to study methodology
Prior evidence for the time at which schizophrenia reaches a plateau during the first 5 years^92^	Duration period reduced to minimise confounding due to variation in disease duration and time on antipsychotic therapy^42,45,52,63,154^
The majority of the deterioration in psychosocial functioning occurs within the first 3 years^93^	Duration period extended to increase the number of eligible participants^169^
Most functional disability occurs within the first 5 years of the disease^164^	Duration period chosen to maintain consistency with another trial^32^
Minimum duration of follow-up for first-episode studies should be 2 years^150^	
Patients with adolescent onset of schizophrenia and duration less than 3 years are believed to be in a ‘critical period’^152^	
Progression of the disease might occur predominantly in the first 5 years^34^	

Finally, seven studies selected patients with disease duration between two periods of time to define early schizophrenia, ranging from 6 months to 7 years in length, starting from 1 month since diagnosis to as late as 24 months thereafter.^35,37,59,74,76,140,176^ Two of these studies had also used ‘less than’ disease duration definitions with cut-offs of <1 year and <2 years, respectively, to define a separate subgroup of schizophrenia patients with earlier disease.^35,74^

#### Symptom severity-based definitions

The review identified 22 relevant studies that incorporated symptom severity-based definitions (e.g. requiring patients to be in an acute phase of the disease or in a stable phase or remission).^30,32,36,37,40,48,67,76–78,100,137,172,173,178–185^ All of these studies used the symptom severity criteria in combination with a duration or episode-based definition. It should be noted that in many of the first-episode studies it was often implied that these patients were in the acute phase of an episode, however as symptom severity was not a formal patient selection criterion these studies were therefore not included under this definition type. One additional study employing the 5-stage model proposed by McGorry et al. to describe the onset of psychiatric disorders for the purposes of selecting patients for the study was identified. This study included patients who fulfilled the criteria for stage 2 (first episode of psychosis).^42^

### Early schizophrenia terminology

The most commonly used terminology for early schizophrenia included ‘first-episode’, ‘recent-onset’, ‘early-phase’, ‘early course’, ‘early’ and ‘early stage’ schizophrenia. Terminology varied somewhat between studies using the duration, episode and/or severity definitions. In particular, the terms ‘first-episode’ and ‘recent-onset’ were more frequently used in studies that included disease duration or severity definitions, whereas studies with multi-episode definitions tended to use terms such as ‘early phase’ or ‘early course’. However, there was no clear association of particular terms with specific definitions.

## Discussion

From the results of this literature review it is clear that a wide variety of definitions are being used to describe patients with early schizophrenia, likely based on the different study types represented in the review and their objectives for patient inclusion. The majority of guidelines used the terminology ‘first-episode psychosis’ when discussing patients early in the course of the disease, while in primary studies, the most commonly used criterion to define early schizophrenia, was for patients to be in their first episode of schizophrenia, followed by application of disease duration limits, frequently disease duration less than 1, 2 or 5 years. Only a small number of studies used multi-episode or symptom severity/staging criteria to select patients with early schizophrenia. The majority of studies did not use a combination of definitions, with many studies simply identifying patients being in their first episode of schizophrenia.

The onset of psychosis has been found to be difficult to determine, as onset may be rapid or more subtle, and may be preceded by months to years of prodromal disease.^186^ Further, although in the majority of cases time of symptom onset is obtained from parents or close relatives, in some cases it may rely on patients’ recollection only, causing additional variability. There were wide variations in definitions used to mark the onset of schizophrenia in studies identified in this review as using duration-based definitions, with a large proportion of studies not specifying when illness or disease was considered to start. Of the studies that did provide a definition, the most common was to refer to time since symptom onset, however no studies provided clear information on how the first occurrence of symptoms was detected. As a result, there may be variation in how the definition was applied between patients, and also between studies. The next most common definitions of onset were first presentation to mental health services, hospitalisation or admission, and first diagnosis. While these may be more objectively measured than symptom onset, by this stage patients have already developed psychotic symptoms, and therefore it could be argued that the onset of schizophrenia occurred some time before this point.

A number of review articles have discussed the validity of using episode-based definitions in schizophrenia, particularly ‘first-episode’ definitions, highlighting difficulties including the lack of a consensus definition of ‘first-episode schizophrenia’,^187,188^ and the fact that first-episode samples may still show great heterogeneity due to potential uncertainty of a schizophrenia diagnosis at the first episode and variation in illness duration.^8,188,189^ Use of multi-episode definitions is further complicated by the need to define when one episode has ended and the next has started. The lack of a standardised definition of ‘relapse’ has been highlighted in a systematic review of observational studies,^190^ and few studies using multi-episode definitions identified in the present review specified a definition for relapse. The majority of studies that did define relapse in this review used hospitalisation as a proxy for identifying it, which may be problematic as patients could be hospitalised for reasons other than a worsening of symptoms and conversely, patients may suffer a worsening of symptoms but not be hospitalised. This is particularly true in countries where crisis management teams have been implemented in the community setting. Other studies used a symptom severity scale as part of the definition for relapse, but there was variation in the scales used as well as the cut-off scores used with those scales. Only one of the studies using multi-episode definitions identified in this review defined how subsequent episodes were measured, defining these as relapses requiring psychiatric hospitalisation.^37^

With regards to definitions using symptom severity, an article by Francey et al. (2010) suggested that use of symptom severity alone may not be sufficient in defining first-episode schizophrenia, particularly when determining whether antipsychotic medications should be used.^189^ Further, duration-based definitions are flawed by limitations such as difficulties in reliably measuring symptom duration, especially retrospective estimation of the duration of untreated psychosis,^191^ as well as heterogeneity between patients, particularly when a long disease duration is allowed.

Although no clear differences in the types of definition used were seen when stratifying studies by study design, a somewhat higher proportion of cross-sectional studies used overlapping definitions, whereas other study designs tended to use single definitions. This may indicate that cross-sectional studies, many of which were imaging studies, tended to require a more homogeneous patient population, whereas for other studies broader definitions may have been more appropriate in order to achieve a larger sample size.

In addition to the lack of consensus in definitions identified in this review, terminology used to refer to ‘early schizophrenia’ is equally heterogeneous. The term ‘early schizophrenia’ itself is problematic since it may be used both to refer to patients early in the course of the disease and to patients who have disease onset at an early age. As a result definitions such as ‘recent onset’, ‘early phase’, ‘early course’ and ‘early stage’, were more commonly used in the literature. As identified from the review of treatment guidelines, the term ‘first-episode psychosis’ is often used in place of ‘first-episode schizophrenia’. This is in part because at the time of presenting with a first psychotic episode it can be challenging to establish a diagnosis of schizophrenia as opposed to other psychiatric diagnoses or other underlying causes.^16,19,26^ An added factor relates to the stigma attached to the term ‘schizophrenia’ which may influence the timing of applying a schizophrenia ‘label’.^192,193^ Consequently, the use of ‘first-episode schizophrenia’ as a term may be more applicable in settings where the definition can be applied retrospectively, for example when reviewing the history of a patient with a confirmed diagnosis of schizophrenia but no longer in their first episode of psychosis.^187^

From this literature review, there does not appear to be a single ‘best’ definition of early schizophrenia. Taking the most commonly used definitions in this review, the most ‘standard’ definition is for patients to be in their first episode of schizophrenia, identified due to a first contact with health services or hospitalisation. For further refinement, the most common additional criterion used was to add a disease duration limit of 1, 2 or 5 years from illness onset.

Establishing a consensus definition of early schizophrenia is important for several reasons. First, when applied in clinical practice it can aid prediction of the outcome of different treatment approaches. For example, it is well established that patients treated earlier have a better prognosis, but that those earlier in the disease course can also be more sensitive to treatment side-effects as they typically have limited prior exposure to antipsychotic agents.^19,23^ In this setting the priority should be on establishing a definition that is both straightforward to apply and clinically meaningful. In the context of clinical studies, establishing a consensus definition depends somewhat on the study design and objectives. For ease of comparing results across studies, the emphasis should be on definitions that can be applied consistently to minimise heterogeneity in the included populations. Thus, we propose that the onset of early schizophrenia should be set as the point at which patients first develop symptoms severe enough to be considered a psychotic episode. Regarding the end of early schizophrenia, we suggest that disease duration of <5 years encompasses previous definitions of the critical period for early intervention.^194^ Within this broad definition, it may be possible to identify further subgroups, taking into account early symptom severity, age of onset and time to treatment, which have all been found to be prognostic factors for schizophrenia outcome.^195–197^ Further investigation of the impact of these factors through trajectory modelling could help refine the definition of early schizophrenia, while a consensus panel on this topic is required to drive forward consistency in the way in which early schizophrenia is defined.

### Limitations of the review

Limitations of this literature review include single-reviewer screening and extraction of articles, restriction of searches to articles published from 2005 onwards and including only articles written in the English language, while no geographical restrictions were applied. The search strategy did not include terms for ‘first-episode psychosis’, which may have resulted in some studies including schizophrenia subgroups within this broader population being missed. Further, the results of this review are limited by use of search terms related to ‘early’, rather than searching for all studies conducted in schizophrenia. While we attempted to mitigate this by running iterative searches to include additional terminology identified through the review, there is the potential for over- or under-representation of the terms identified in the results depending on whether they were used as search terms themselves. Additionally, the lack of clarity provided in some publications regarding diagnoses may mean that some studies with mixed schizophrenia disorder populations were inadvertently included in the review. For example, studies using the term ‘DSM-IV for schizophrenia’ despite later referring to a mixture of schizophrenia, schizoaffective disorder and schizophreniform patients were excluded, whereas those simply stating ‘DSM-IV for schizophrenia’ were included.

The focus on definitions used for participants with diagnosed schizophrenia in itself means that findings from this review cannot be extrapolated to broader populations diagnosed with schizophrenia spectrum disorders. Given the difficulty in establishing a diagnosis at the early stages of schizophrenia, there may be value in a broader review of the literature in this expanded population. Nevertheless, since there are differences in the interventions that are licensed for patients diagnosed with schizophrenia versus other schizophrenia spectrum disorders, a review of definitions to guide therapy in this specific population is still relevant.

Finally, determination of the frequency of definitions used was limited by weighting each included study equally, irrespective of population number. A series of small studies using the same definition may thus have had a higher weighting than a single larger study.

## Conclusions

A wide variety of definitions have been used to identify patients with early schizophrenia, with no apparent consensus. First-episode schizophrenia is the most frequently reported terminology in studies of patients with early schizophrenia, in line with the clinical guidelines, but even this common terminology is accompanied by a variety of definitions, including disease duration or symptom severity, and neither the terminology nor the definitions used are without limitations. When not referring specifically to first-episode patients, ‘recent-onset’ was the next most frequently used term, while the duration-based definitions most frequently used duration less than 1, 2 or 5 years. Better agreement on the definition of early schizophrenia could aid interpretation and comparison of studies in this patient population and consensus on definitions should allow for better identification and management of schizophrenia patients in the early course of their disease.

## Methods

### Search strategy and selection of studies

For this targeted literature review, searches for published studies, clinical trial entries and guidelines were conducted between 14.08.2015 and 21.10.2015 using MEDLINE and MEDLINE In-Process (via PubMed), the Australian New Zealand Clinical Trials Registry, ClinicalTrials.gov, the EU Clinical Trials Register, the International Standard Randomised Controlled Trial Number database, and Google (Data Supplement Table 1–7). Searches for published studies and clinical trial records were conducted iteratively, adding in any new terminology for ‘early schizophrenia’ from identified studies as new search terms, until no further new terminology was identified. The searches for relevant guidelines were updated in May 2016.

Studies were eligible for inclusion (Data Supplement Table 8) if they were written in the English language and conducted in humans, included a distinct population of patients with early schizophrenia or discussed definitions of early schizophrenia, and were published from January 1st 2005. Case studies were excluded, but there was no other restriction on study design. No limits were applied regarding interventions, comparators or outcomes. Articles were reviewed against the eligibility criteria by a single reviewer. Where the applicability of the eligibility criteria was unclear, the article was assessed by a second reviewer.

### Data extraction and analysis

Information extracted from relevant studies relating to the search objectives was input into pre-specified Excel workbooks. Data extraction was performed by a single individual for each included study and any uncertainties were reviewed by a second individual.

## Electronic supplementary material

Supplementary Information

## Data Availability

No datasets were generated or analysed during the current study.

## Electronic supplementary material

**Supplementary information** accompanies the paper on the *npj Schizophrenia* website (10.1038/s41537-018-0063-7).
