# Supplementary material for: Diverse definitions of the early course of schizophrenia—a targeted literature review
Source: NPJ Schizophr. 2018 Oct 15;4:21. doi: 10.1038/s41537-018-0063-7 (PMC6189105; doi:10.1038/s41537-018-0063-7)
Supplement: Supplementary file 1 — Supplementary Information [file 41537_2018_63_MOESM1_ESM.pdf]

Diverse Definitions of the Early Course of Schizophrenia – A Targeted Literature Review

SUPPLEMENTARY DATA

Table 1 MEDLINE and MEDLINE In-Process search terms for the definition of early schizophrenia (terms searched simultaneously via the PubMed platform; Round 1)

Date conducted: 14/08/2015			
**Term group**	**Search number**	**Search terms**	**No. hits**
**Early stage schizophrenia**	1	“early schizophrenia”	155
	2	Schizophrenia[tiab] AND ("early-stage" OR "early stages")	553
	3	Schizophrenia AND (“early-phase” OR “early phases”)	267
	4	Early AND “disease course” AND schizophrenia	24
	5	“first-episode schizophrenia”	1230
	6	“first-admission schizophrenia”	16
	7	“drug-naïve schizophrenia”	44
	8	“medication-naïve schizophrenia”	5
	9	“treatment-naïve schizophrenia”	7
	10	“premorbid schizophrenia”	10
	11	“prodromal schizophrenia”	21
	12	“preonset schizophrenia”	3
	13	“preprodromal schizophrenia”	1
**Publication Date**	14	“2005/01/01”[PDAT] : “3000/12/31”[PDAT]	9014414
**Total**	15	OR/1-13	2163
	16	14 AND 15	1559

Table 2 Searches conducted in clinical trial databases (Round 1)

Database	Search terms	Date	No. hits
**ClinicalTrials.gov**	**Terms:** young OR early OR "first episode" OR "first-episode" OR "first-admission" OR "first admission" OR naive OR premorbid OR prodromal OR "pre-onset" OR preonset OR preprodromal<br><br>**Condition:** schizophrenia	20/08/2015	340
**ISRCTN**	**Condition:** schizophrenia	08/09/2015	128
**ANZCTR**	**Terms:** schizophrenia	12/09/2015	90
**EU CTR**	**Terms:** schizophrenia AND (early OR "first-episode" OR "first episode" OR "first-admission" OR "first admission" OR "drug-naive" OR "drug naive" OR "medication-naive" OR "medication naive" OR "treatment-naive" OR "treatment-naive" OR "premorbid" OR "prodromal" OR "pre-onset" OR "preonset" OR "preprodromal" OR young)	12/09/2015	55

Table 3 MEDLINE and MEDLINE In-Process search terms for the definition of early schizophrenia (terms searched simultaneously via the PubMed platform; Round 2)

**Date conducted: 22/09/2015**			
**Term group**	**Search number**	**Search terms**	**No. hits**
**Early stage schizophrenia**	1	"early course" AND "schizophrenia"	192
**Publication Date**	2	"2005/01/01"[PDAT] : "3000/12/31"[PDAT]	9157401
**Deduplication against prior searches**	3	(("2005/01/01"[PDAT] : "3000/12/31"[PDAT]) AND ((((((((((("early schizophrenia") OR ((Schizophrenia[tiab] AND ("early-stage" OR "early stages")))) OR (OR (Schizophrenia AND ("early-phase" OR "early phases"))) OR ((Early AND "disease course" AND schizophrenia))) OR "first-episode schizophrenia") OR "first-admission schizophrenia") OR "drug-naive schizophrenia") OR "medication-naive schizophrenia") OR "treatment-naive schizophrenia") OR "premorbid schizophrenia") OR "prodromal schizophrenia") OR "preonset schizophrenia") OR "preprodromal schizophrenia"))	1592
**Total**	4	1 AND 2	121
	5	4 NOT 3	79

Table 4 MEDLINE and MEDLINE In-Process search terms for the definition of early schizophrenia (terms searched simultaneously via the PubMed platform; Round 3)

**Date conducted: 23/09/2015**			
**Term group**	**Search number**	**Search terms**	**No. hits**
**Early stage schizophrenia**	1	"post-acute" AND "schizophrenia"	40
**Publication Date**	2	"2005/01/01"[PDAT] : "3000/12/31"[PDAT]	9159726
**Deduplication against prior searches**	3	((((("early phases" OR "early phase") AND schizophrenia) AND ("2005/01/01"[PDAT] : "3000/12/31"[PDAT]))) OR (("2005/01/01"[PDAT] : "3000/12/31"[PDAT]) AND ((((((((((("early schizophrenia") OR ((Schizophrenia[tiab] AND ("early-stage" OR "early stages")))) OR ((Early AND "disease course" AND schizophrenia))) OR "first-episode schizophrenia") OR "first-admission schizophrenia") OR "drug-naive schizophrenia") OR "medication-naive schizophrenia") OR "treatment-naive schizophrenia") OR "premorbid schizophrenia") OR "prodromal schizophrenia") OR "preonset schizophrenia") OR "preprodromal schizophrenia")))) OR ((((("2005/01/01"[PDAT] : "3000/12/31"[PDAT]) AND "early course" AND "schizophrenia"))))	1672
**Total**	4	1 AND 2	16
	5	4 NOT 3	13

Table 5 MEDLINE and MEDLINE In-Process search terms for the definition of early schizophrenia (terms searched simultaneously via the PubMed platform; Round 4)

**Date conducted: 12/10/2015**			
**Term group**	**Search number**	**Search terms**	**No. hits**
**Early stage schizophrenia**	1	("recent-onset" AND and schizophrenia) OR (postdrom* AND and schizophrenia)	361
**Publication Date**	2	"2005/01/01"[PDAT] : "3000/12/31"[PDAT]	9209779
**Deduplication against prior searches**	3	((((((((("early phases" OR "early phase") AND schizophrenia) AND ("2005/01/01"[PDAT] : "3000/12/31"[PDAT]))) OR (("2005/01/01"[PDAT] : "3000/12/31"[PDAT]) AND ((((((((((("early schizophrenia") OR ((Schizophrenia[tiab] AND ("early-stage" OR "early stages")))) OR ((Early AND "disease course" AND schizophrenia))) OR "first-episode schizophrenia") OR "first-admission schizophrenia") OR "drug-naive schizophrenia") OR "medication-naive schizophrenia") OR "treatment-naive schizophrenia") OR "premorbid schizophrenia") OR "prodromal schizophrenia") OR "preonset schizophrenia") OR "preprodromal schizophrenia"))))) OR ((((("2005/01/01"[PDAT] : "3000/12/31"[PDAT]) AND "early course" AND "schizophrenia"))))) OR (((((((("2005/01/01"[PDAT] : "3000/12/31"[PDAT]) AND "post-acute" AND "schizophrenia")))))))))	1695
**Total**	4	1 AND 2	231
	5	4 NOT 3	181

Table 6 MEDLINE and MEDLINE In-Process search terms for the definition of early schizophrenia (terms searched simultaneously via the PubMed platform; Round 5)

**Date conducted: 12/10/2015**			
**Term group**	**Search number**	**Search terms**	**No. hits**
**Early stage schizophrenia**	1	"new-onset schizophrenia"	10
**Publication Date**	2	"2005/01/01"[PDAT] : "3000/12/31"[PDAT]	9209779
**Deduplication against prior searches**	3	((((((((("early phases" OR "early phase") AND schizophrenia) AND ("2005/01/01"[PDAT] : "3000/12/31"[PDAT]))) OR (("2005/01/01"[PDAT] : "3000/12/31"[PDAT]) AND ((((((((((("early schizophrenia") OR ((Schizophrenia[tiab] AND ("early-stage" OR "early stages")))) OR ((Early AND "disease course" AND schizophrenia))) OR "first-episode schizophrenia") OR "first-admission schizophrenia") OR "drug-naive schizophrenia") OR "medication-naive schizophrenia") OR "treatment-naive schizophrenia") OR "premorbid schizophrenia") OR "prodromal schizophrenia") OR (("recent-onset" AND and schizophrenia) OR (postdrom* AND and schizophrenia)) OR "preonset schizophrenia") OR "preprodromal schizophrenia"))))) OR ((((("2005/01/01"[PDAT] : "3000/12/31"[PDAT]) AND "early course" AND "schizophrenia"))))) OR (((((((("2005/01/01"[PDAT] : "3000/12/31"[PDAT]) AND "post-acute" AND "schizophrenia")))))))))	1877

**Total**	4	1 AND 2	7
	5	4 NOT 3	6

Table 7 Searches conducted in clinical trial databases (Round 2)

Database	Search terms	Date	No. hits
**ClinicalTrials.gov**	**Terms:** "recent onset" OR "recent-onset" OR "early course" OR "post-acute" OR "post acute" OR "early-course" OR "post-dromal" OR postdromal OR "new onset" OR "new-onset"<br><br>**Condition:** schizophrenia	21/10/2015	39
**ISRCTN**	N/A		
**ANZCTR**	N/A		
**EU CTR**	**Terms:** schizophrenia AND ("recent onset" OR "recent-onset" OR "early course" OR "post-acute" OR "post acute" OR "early-course" OR "post-dromal" OR postdromal OR "new onset" OR "new-onset")	21/10/2015	4

Table 8 Inclusion criteria for the definition of early schizophrenia review

Domain	Description
**Population**	Patients with early schizophrenia. Only 'pure' populations of early schizophrenia patients were included; studies recruiting patients with any type of schizophrenia spectrum disorder (e.g. schizophreniform disorder) were not included
**Intervention**	No inclusion criteria regarding interventions
**Comparator**	No inclusion criteria regarding comparators
**Outcomes**	Any outcome as long as the study reported a definition of early schizophrenia
**Study design**	Case studies were excluded, but there was no other restriction on study design
**Other considerations**	• Publications were required to be full-texts or abstracts written in the English language<br>• Articles were required to discuss relevant definitions or include a relevant definition within their eligibility criteria<br>Relevant definitions were those relating to patients diagnosed with schizophrenia (e.g. DSM-IV codes 295.10, 295.20, 295.30, 295.60, 295.90; ICD-10 codes F20.0–F20.9) and experiencing more than one psychotic episode, fulfilling particular symptom severity criteria (e.g. being in an acute or stable phase), or with a specified duration of illness (e.g. less than 5 years)
